# Prognosis of rapid onset functional tic‐like behaviors: Prospective follow‐up over 6 months

**DOI:** 10.1002/brb3.2606

**Published:** 2022-05-20

**Authors:** Megan Howlett, Davide Martino, Christelle Nilles, Tamara Pringsheim

**Affiliations:** ^1^ Department of Psychiatry University of Calgary Calgary Canada; ^2^ Department of Clinical Neurosciences, Cumming School of Medicine University of Calgary Calgary Canada; ^3^ Mathison Centre for Mental Health Research and Education Calgary Canada; ^4^ Hotchkiss Brain Institute University of Calgary Calgary Canada; ^5^ Department of Clinical Neurosciences, Psychiatry, Pediatrics and Community Health Sciences University of Calgary Calgary Canada

**Keywords:** COVID‐19 pandemic, functional tic‐like behaviors, prognosis, tics

## Abstract

**Background and Purpose:**

The prognosis of rapid onset functional tic‐like behaviors (FTLBs) is unknown. This prospective cohort study describes the course and treatment of rapid onset FTLBs in adolescents (*n* = 20) and adults (*n* = 9) previously reported in two case series.

**Methods:**

Yale Global Tic Severity Scale (YGTSS) scores were compared between first clinical presentation and 6‐month follow‐up assessment. All treatments used for FTLBs and any psychiatric comorbidities were recorded.

**Results:**

In adolescents with FTLBs, motor tics, vocal tics, total tics, impairment, and global scores on the YGTSS significantly improved at 6 months, with a mean decrease in the YGTSS global score of 31.9 points, 95% confidence interval (CI) 15.4, 48.4, *p* = .0005. In adults with FTLBs, only impairment and global scores significantly improved, with a mean decrease in the YGTSS global score of 19.6 points, 95% CI −3.2, 42.3, *p* = .04. Selective serotonin reuptake inhibitors (SSRIs) and cognitive behavioral therapy (CBT) for anxiety and depression were the most used treatment in both age groups.

**Conclusions:**

This prospective study suggests that adolescents have a better prognosis than adults with FTLBs. Management of comorbidities with SSRIs and CBT seems effective.

## Graphical abstract

This prospective cohort study describes the course and treatment of rapid onset functional tic like behaviours (FTLBs) in adolescents (n = 20) and adults (n = 9) over 6 months. In adolescents, total tics and overall impairment significantly improved at six months. In adults, only impairment significantly improved. SSRIs and cognitive behavioural therapy (CBT) for anxiety and depression were the most used treatment in both age groups.

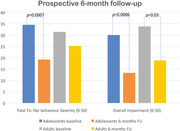

## 1 INTRODUCTION

Since the onset of the SARS‐CoV‐2 pandemic, some centers have reported an increase in referrals for patients with functional neurological disorders (FND) (Hull et al., 2021). During this period, clinicians have become increasingly aware of a rising phenomenon of functional tic‐like behaviors (FTLBs) which predominantly occur in adolescents and young adults (Buts et al., 2021; Müller‐Vahl et al., 2021; Pringsheim & Martino, 2021; Pringsheim et al., 2021) and represent a distinct subtype of FND. The majority of these patients, especially in North America, have been noted to be female (Buts et al., 2021; Pringsheim & Martino, 2021; Pringsheim et al., 2021), whereas one sample in Germany was more equal in gender distribution (Müller‐Vahl et al., 2021). There are notable differences between the symptomatology, age of onset, and course of illness with FTLBs versus Tourette syndrome (TS) or other primary tic disorders (Buts et al., 2021; Müller‐Vahl et al., 2021; Pringsheim & Martino, 2021; Pringsheim et al., 2021). With FTLBs, patients commonly exhibit rapid onset of symptoms (with escalation to peak severity within hours to days) (Buts et al., 2021; Müller‐Vahl et al., 2021; Pringsheim & Martino, 2021; Pringsheim et al., 2021), later age of onset, and complex motor and vocal tic‐like behaviors that do not follow the expected rostrocaudal nor simple to complex progression typically seen in TS (Buts et al., 2021; Müller‐Vahl et al., 2021; Pringsheim & Martino, 2021; Pringsheim et al., 2021). Patients with FTLBs present with higher Yale Global Tic Severity Scale (YGTSS) total tic and impairment scores than patients with TS and are more likely to meet the clinical threshold for an anxiety disorder or major depressive disorder (Müller‐Vahl et al., 2021; Pringsheim & Martino, 2021; Pringsheim et al., 2021). Many of the young people when initially studied were unable to attend school or work nor perform some of their activities of daily living (Pringsheim et al., 2021).

Theories for such magnification of FTLBs in clinical settings include the possibility of social contagion illnesses spread through the medium of social media (Buts et al., 2021; Müller‐Vahl et al., 2021; Pringsheim & Martino, 2021; Pringsheim et al., 2021). For example, many individuals presented with complex vocalizations, including the common expressions and coprolalia displayed by social media influencers, which is concerning for an aspect of social contagion (Müller‐Vahl et al., 2021; Pringsheim & Martino, 2021; Pringsheim et al., 2021). Additional stressors through the pandemic, including school and extracurricular activity closures, “virtual schooling” and associated academic pressures, and increased tensions at home during lockdown measures are also thought to contribute to this clinical phenomenon (Buts et al., 2021; Canadian Mental Health Association, 2020; Pringsheim & Martino, 2021; Pringsheim et al., 2021). For adults, job insecurity, heightened substance use, social isolation, and increased domestic violence may be implicated in experienced mental health symptoms throughout the pandemic (Canadian Mental Health Association, 2020). The COVID‐19 pandemic has heightened mental health symptoms, such as anxiety, and the demand for associated services (Canadian Mental Health Association, 2020; Daly & Robinson, 2021; Rossi et al., 2021; Vigo et al., 2021; Wong et al., 2021).

In 2021, we published two case series of 20 adolescents and nine adults with rapid onset FTLBs seen in Calgary before the end of June 2021 (Pringsheim & Martino, 2021; Pringsheim et al., 2021). The purpose of this prospective cohort study is to report the trajectory and course of illness after 6 months of follow‐up of these originally reported cases, as well as their response to medical and behavioral interventions. It was hypothesized that patients with FTLBs would improve at 6‐month follow‐up with behavioral interventions and treatment of comorbid mental health concerns.

## 2 METHODS

All 29 patients reported in our two case series were diagnosed by one of two movement disorders neurologists (Tamara Pringsheim or Davide Martino), and were offered continuing care at our center. Our overall treatment approach for FTLBs included medical and behavioral treatment for co‐occurring anxiety and depression, in addition to the Comprehensive Behavioral Intervention for Tics (CBIT). At the initial consultation, the Yale Global Tic Severity Scale (YGTSS) was completed, and this was repeated in all patients returning at 6 months. At the 6‐month assessment, all treatments used for FTLBs and any psychiatric comorbidities were recorded. A paired *t*‐test was used to compare YGTSS scores at initial presentation and follow‐up. This project received ethical approval from the University of Calgary Conjoint Health Research Ethics Board. Subjects gave informed consent for participation.

## 3 RESULTS

### 3.1 Adolescent sample

Of the 20 adolescent cases, 19 were assigned female at birth and one was assigned male at birth. Eleven identified as cis‐gender, nine identified as trans, nonbinary, or gender fluid. Mean age at first clinical visit was 14.3 years (95% confidence interval [CI] 13.5, 15.0). Comorbid Attention Deficit Hyperactivity Disorder (ADHD) affected 25%, Obsessive Compulsive Disorder (OCD) 5%, Generalized Anxiety Disorder or Panic Disorder 75%, and Depression 55%. None of the adolescents had a past medical history of FND.

Fifteen adolescents returned for follow‐up at 6 months. Of the five who did not return, one stated that they had no further concerns about tics, and four did not attend phone calls offering follow‐up. There was a significant improvement from initial consultation to 6‐month follow‐up in motor tics, vocal tics, total tics, impairment, and global score on the YGTSS, with a mean decrease of 31.9 points on the YGTSS global score (95% CI 15.4, 48.4, *p* = .0005) (see Table 1). Seven adolescents had YGTSS impairment scores of zero at follow‐up. Two adolescents had YGTSS global scores of zero, and four had YGTSS global scores less than 10.

**TABLE 1:** Tic severity scores

Adolescent sample (*n* = 15)	Initial consultation	6‐month follow‐up visit	Mean change from initial consultation to 6‐month follow‐up	*p*‐Value
YGTSS total motor tic score	17.7 (15.3, 20.2)	10.4 (6.5, 14.3)	−7.3 (−11.0, −3.7)	*p* = .0004
YGTSS total vocal tic score	16.7 (13.2, 20.3)	8.8 (4.1, 13.5)	−7.9 (−12.7, −3.2)	*p *= .0015
YGTSS total tic score	34.5 (28.6, 40.3)	19.2 (10.8, 27.6)	−15.3 (−23.5, −7.0)	*p* = .0007
YGTSS impairment score	30.0 (23.4, 36.6)	13.3 (4.8, 21.9)	−16.7 (−25.5, −7.9)	*p* = .0006
YGTSS global score	64.5 (52.3, 76.6)	32.5 (16.0, 49.1)	−31.9 (−48.4, −15.4)	*p* = .0005

Adult sample (*n* = 9)	Initial consultation	6‐month follow‐up visit	Mean change from initial consultation to 6‐month follow‐up	*p*‐Value
YGTSS total motor tic score	16.9 (13.7, 20.1)	14.0 (8.2, 19.8)	−2.9 (−8.1, 2.3)	*p* = .12
YGTSS total vocal tic score	14.6 (10.2, 18.9)	11.2 (5.6, 16.9)	−3.3 (−8.5, 1.9)	*p* = .09
YGTSS total tic score	31.4 (24.5, 38.4)	25.2 (14.5, 35.9)	−6.2 (−16.4, 3.9)	*p* = .1
YGTSS impairment score	32.2 (21.5, 42.9)	18.8 (5.9, 31.9)	−13.3 (−27.7, 1.0)	*p* = .03
YGTSS global score	63.7 (47.7, 79.6)	44.1 (21.0, 67.2)	−19.6 (−42.3, 3.2)	*p* = .04

*Note*: Mean and 95% confidence intervals are given in brackets.

Abbreviation: YGTSS, Yale Global Tic Severity Scale.

The most used treatments were selective serotonin reuptake inhibitors (SSRIs) and cognitive behavioral therapy (CBT) for anxiety or depression (Table 2). One adolescent was taking an antipsychotic medication (aripiprazole) for treatment augmentation for depression. Both adolescents with YGTSS global scores of zero were receiving an SSRI (sertraline, fluoxetine) and CBT for anxiety or depression. Two adolescents developed psychogenic nonepileptic seizures (PNES) during the follow‐up period. No other functional neurological symptoms were seen at follow‐up.

**TABLE 2:** Treatments at 6‐month follow‐up

Treatment	Adolescent sample (*n* = 15)	Adult sample (*n* = 9)
Alpha agonists	3 (20%)	0
Antipsychotics	1 (7%)	3 (33%)
SSRIs	7 (47%)	3 (33%)
Non‐SSRI antidepressant	1 (7%)	1 (11%)
Psychostimulants	2 (13%)	0
Comprehensive behavioral intervention for tics	3 (20%)	2 (22%)
Cognitive behavioral therapy for anxiety or depression	8 (53%)	4 (44%)

Abbreviation: SSRIs, selective serotonin reuptake inhibitors.

### 3.2 Adult sample

Of the original reported nine adult cases, eight were assigned female at birth and one was assigned male at birth. Seven identified as cis‐gender, and two identified as trans. Mean age at first clinical visit was 19.9 (95% CI 18.8, 20.0). Comorbid ADHD affected 22%, OCD 0%, Generalized Anxiety Disorder or Panic Disorder 56%, and Depression 44%. None of the adults had a past medical history of FND. One adult patient presented with episodic functional leg weakness concurrently with FTLBs.

All nine adults returned for follow‐up at 6 months. While there was a decrease in motor tics, vocal tics, and total tics on the YGTSS from initial consultation to 6‐month follow‐up, the change was not statistically significant (Table 1). Impairment and global scores on the YGTSS were significantly lower at 6 months, with a mean decrease on the global score of 19.6 points (95% CI −3.2, 42.3, *p* = .04). Three adults had YGTSS impairment scores of zero at follow‐up, and one had a YGTSS global score of zero.

The most commonly used treatments over the 6‐month period of follow‐up were antipsychotics, SSRIs, and CBT for anxiety or depression (Table 2). Of the three adults taking antipsychotics, two were taking quetiapine and one was taking aripiprazole, all for augmentation for depression. One adult with a YGTSS global score of zero at follow‐up was receiving sertraline and quetiapine, and CBT for anxiety. Two adults developed PNES during the follow‐up period. No other functional neurological symptoms were seen at follow‐up.

## 4 DISCUSSION

Adolescents had a better outcome than adults with FTLBs, which is consistent with previous literature in FNDs. In one study, forty adolescents with FND assessed and treated at a child psychiatry referral center were re‐evaluated 4 years after their initial presentation. Children received treatment for FND symptoms and comorbid psychiatric disorders. Eighty‐five percent were found to have completely recovered, 5% had improved, and only 10% were unchanged. Mood and anxiety symptoms continued to occur at a considerable rate despite recovery from FND symptoms (Pehlivantürk & Unal, 2002). Similarly, a study documenting the 12‐month outcome of FND in 147 under 16 years old found that symptoms were improved in over 90% of children with motor weakness, abnormal movements, paralysis, and nonepileptic seizures (Ani et al., 2013). Twenty‐eight percent were diagnosed with a new psychiatric disorder during the follow‐up period, suggesting progress away from somatic to emotional expression of distress in some children. Psychological interventions were offered and accepted by the majority of patients, including psychoeducation, supportive counselling, anxiety management, family therapy, and cognitive behavioral therapy. In contrast, adult studies suggest a lower rate of symptom resolution. Persistence of FND was found in 90% of adults at an average follow‐up of 3.2 years (Feinstein et al., 2001) in a Canadian study, despite 74% of patients receiving psychiatric care after the FND diagnosis was made. Another study reported resolution or improvement in 58% of adults with medically unexplained motor symptoms after 6 years of follow‐up (Crimlisk et al., 1998). Medical and psychological treatment for this sample was not described.

In children and adolescents, FNDs have shown improvement with multidisciplinary cognitive and behavioral treatment, with greater efficacy in those with a shorter time from symptom onset to diagnosis and treatment (Schwingenschuh et al., 2008). Factors that have been posited for favorable outcomes in children and adolescents included early diagnosis and good premorbid adjustment (Pehlivantürk & Unal, 2002). Similar findings have been reported in adults whereby poor outcomes occurred in those with longer symptom duration, insidious onset, inability to acknowledge the psychological nature of their condition, and psychiatric comorbidity. Others have found strong physical health, positive social life perceptions, perception of effective treatment, elimination of stressors, and treatment with medication were positive indicators of a favorable outcome in adults (Thomas et al., 2006). It is possible that advocacy from parents leads to more rapid assessment and management of FNDs for adolescents, improving prognosis.

We have seen approximately 60 additional cases of rapid onset FTLBs at our site since the publication of our two case series and have had treatment success in both adolescents and adults using a combination of SSRIs and CBT to target comorbid anxiety and depressive symptoms. We believe that the functional behavioral assessment and intervention component of the Comprehensive Behavioral Intervention for Tics (CBIT) could be an effective treatment strategy for FTLBs, but few of our patients have been able to access this therapy as it is not publicly funded. As part of the functional behavioral assessment and intervention, we discuss the importance of self‐awareness of triggers for FTLBs, including social media use, and managing exposure if this is an important exacerbating factor.

Our study bears limitations. Five of our adolescents are currently lost to follow‐up. We suspect that these are more likely individuals with a favorable prognosis, as patients with more severe symptoms should be more likely to seek specialist monitoring, and this may lead to underestimation of the positive short‐term outcome. However, the reverse may also be true—patients unwilling to accept the diagnosis of FND may refuse follow‐up visits. This study focused on short‐term outcomes but did not delve into a comprehensive analysis of outcome predictors due to the small sample size. Ongoing research at our institution is exploring the association of social and adaptive functioning factors with diagnosis and prognosis of FTLBs with onset during the SARS‐CoV‐2 pandemic.

## CONFLICT OF INTEREST

Davide Martino has received compensation for consultancies for Sunovion and Merz Pharmaceuticals, honoraria from Movement Disorders Society, Dystonia Medical Research Foundation Canada and Merz Pharmaceuticals, royalties from Springer‐Verlag, research support from Ipsen Corporate, funding grants from: Dystonia Medical Research Foundation Canada, Parkinson Canada, The Owerko Foundation, and the Michael P Smith Family. Tamara Pringsheim has received research funding from the Public Health Agency of Canada, the Maternal Newborn Child and Youth Strategic Clinical Network of Alberta Health, and the Owerko Centre of Alberta Children's Hospital Research Institute. Christelle Nilles and Megan Howlett declare no conflict of interst.

## AUTHOR CONTRIBUTIONS

*Study execution, writing, and editing*: Megan Howlett. *Study design, execution, writing, and editing*: Davide Martino. *Study execution, writing, and editing*: Tamara Pringsheim. *Study design, execution, writing, and editing*: Christelle Nilles.

### PEER REVIEW

The peer review history for this article is available at https://publons.com/publon/10.1002/brb3.2606.

## Data Availability

The data that support the findings of this study are available on request from the corresponding author. The data are not publicly available due to privacy or ethical restrictions.
